# Telemedicine Consultations: An Alternative Model to Increase Access to Diabetes Specialist Care in Underserved Rural Communities

**DOI:** 10.2196/resprot.2235

**Published:** 2012-11-07

**Authors:** Frederico G Toledo, Amy Triola, Kristine Ruppert, Linda M Siminerio

**Affiliations:** 1 Div. of Endocrinology and MetabolismDept. of MedicineUniversity of PittsburghPittsburgh, PAUnited States; 2 University of Pittsburgh Medical CenterPittsburgh, PAUnited States; 3 Graduate School of Public HeatlhDept. of EpidemiologyUniversity of PittsburghPittsburgh, PAUnited States

**Keywords:** Rural, teleconsultation, telemedicine, diabetes

## Abstract

**Background:**

Diabetes care in rural communities often suffers because of physician shortages. When patients need to see an endocrinologist, long-distance travel to urban centers can constitute a barrier to care.

**Objective:**

To address this problem, we tested whether diabetes telemedicine consultations would be acceptable to rural patients and their primary care providers as an alternative care model.

**Methods:**

Twenty-five patients with diabetes in a rural, medically underserved community received glycemic management recommendations via videoconferencing-based teleconsultation with an endocrinologist at an urban center. At the rural site, a nurse trained in diabetes care assisted with the visits. Outcomes measured were patient and primary care provider satisfaction (measured by structured questionnaires) and glycosylated hemoglobin (HbA1c) levels.

**Results:**

Patients and providers uniformly reported high levels of satisfaction and acceptability. Mean HbA1c decreased from 9.6% to 8.5% (*P* < .001).

**Conclusions:**

Teleconsultations are well accepted by users (patients and primary care physicians) and glycemic control seems to improve in patients with diabetes. This new model of care could potentially expand access to specialist care in isolated rural communities.

## Introduction

Rural communities in the United States suffer from a disproportionate burden of diabetes and lower quality of diabetes care than metropolitan areas [1-3]. In medically underserved rural communities, primary care providers who care for challenging cases of poorly controlled diabetes may encounter difficulties in supporting optimal diabetes care and, thus, meeting quality of care standards. Shortages of endocrinologists can complicate this problem [4]. Patients in isolated rural areas often need to travel long distances to establish care with an endocrinologist, often located in urban areas. The travel time and expense associated with transportation can be major barriers to medical care. Therefore, in order to improve the quality of diabetes care in rural, medically underserved communities, newer models of care should take into account the problem of physical separation between specialist provider and patient.

The emerging field of telemedicine has great potential to mitigate this problem by obviating geographical barriers to care. Advances in videoconferencing now make it possible to extend diabetes expertise to rural communities, thus helping patients and primary care providers. However, diabetes mellitus has its own complexities of care requiring comprehensive patient education, especially in regards to starting and adjusting insulin therapy, education on glucose monitoring, and self-management skills. Implementation of these types of recommendations is pragmatically more difficult via videoconferencing when compared to implementation in-person at the office. Therefore, we developed a new model of care that includes teleconsultations provided by a remotely located endocrinologist combined with diabetes self-management education provided locally at the rural site by a nurse trained in diabetes care. This is a new model and acceptability has not been demonstrated yet. To our knowledge, previous studies of telemedicine have not specifically combined diabetes advice by an endocrinologist with diabetes education provided locally at the rural office. Furthermore, the acceptability of this model must be specifically demonstrated in rural communities, which have their own particular demographics and barriers to care. Likewise, acceptability by primary care providers in rural areas must be demonstrated to better understand implications for future referrals. Acceptability studies are needed before resources are committed to large-scale programs or expanding telecommunications infrastructure. To address these gaps, we conducted a study in an isolated rural community to demonstrate the acceptability of a novel diabetes teleconsultation model to optimize glycemia.

## Methods

### Recruitment

Protocols were approved by the University of Pittsburgh Institutional Review Board. Research participants gave written informed consent. Patients were recruited between July and October 2009. Patients (n = 25) and primary care providers (n = 7) were in a rural, medically underserved community in Pennsylvania located approximately 95 miles from the nearest endocrinology referral center. Seven primary care providers were contacted and agreed to participate, receiving no participation incentives or compensation to avoid bias. Primary care providers were notified of the study and invited to refer patients with diabetes in whom previous attempts to improve glycemic control had failed under their care. Primary care providers referred the patient participants to the study team nurse who scheduled a subsequent appointment for teleconsultation.

### Procedures and Analysis

Each participant had a one-time teleconsultation (45 minutes) aimed at troubleshooting hyperglycemia. The teleconsultation videoconferencing equipment used was the Polycom HDX 4002 system (20-inch high-definition widescreen operating at 720 lines/progressive scan) located in an endocrinology office in urban Pittsburgh, Pennsylvania (hub), and in a rural clinic office (rural site). These sites were connected via a dedicated broadband Internet connection. Embedded Advanced Encryption Standard (AES) Federal Information Processing Standard (FIPS) 197, H.235v3 and H.233/234 encryption was used to protect the confidentially of the data transmission.

An endocrinologist (hub) and diabetes nurse (rural site) operated the videoconferencing equipment. The trained diabetes nurse worked as a liaison during the visit, ensuring coordination of care between sites. Teleconsultations included a medical interview and laboratory data review, followed by treatment recommendations carried out with the assistance of the nurse. Recommendations regarding the management plan (medication adjustments, lifestyle modification, self-monitoring, and laboratory tests) were forwarded to the primary care providers by letter. Glycosylated hemoglobin (HbA1c) measurements were obtained from chart review. Patient and provider program satisfaction were measured using researcher-designed satisfaction questionnaires adapted from validated satisfaction surveys in the literature [5,6]. Answers were graded on a Likert scale.

## Results

### Recruitment

Mean patient age was 56 years (range 35-80). Most (24/25, 96%) had Type 2 diabetes. Mean disease duration was 16 years (range 1 month to 41years). All patients (25/25, 100%) were white or Caucasian and 56% (14/25) were women. Although more than half of patients (14/25, 56%) had previously received a recommendation to consult with an endocrinologist, only 24% (6/25) had followed up on that recommendation, reflecting local underutilization of specialists. The distance between hub and rural sites was approximately 95 miles by car travel, but only 40% (10/25) of respondents were willing to travel that distance, demonstrating that travel was a barrier to care in the targeted population.

### Outcomes

Patient satisfaction responses reflected uniform enthusiasm on several dimensions tested (Table 1). There were no instances of equipment or data transmission malfunction during a total of 18.8 hours of videoconferencing. Primary care provider satisfaction also was notably positive. Answers about coordination of care and the clinical value of consults were generally consistent when questions were asked in different ways.

**Table 1:** Patient and provider satisfaction with diabetes teleconsultations.

Questions			Responses
**Access to care, n (%)**			
	**Have you ever been told that you should see an endocrinologist (diabetes specialist)?**		
		Yes	14 (56%)
		No	11 (44%)
	**Have you ever had a visit with an endocrinologist?**		
		Yes	6 (24%)
		No	19 (76%)
	**If your doctor asked you to see an endocrinologist, how far would you be willing to travel to see the specialist?**		
		Unable to travel	1 (4%)
		1-10 miles	7 (28%)
		10-50 miles	7 (28%)
		“Distance does not matter to me”	10 (40%)
**Patient satisfaction, median (interquartile range)** ^**a**^			
	How satisfied were you with your endocrinologist visit through videoconferencing, for example, were you able to communicate well and ask questions during this visit?		6 (6-6)
	How satisfied were you with the videoconference method for a visit with the endocrinologist?		6 (4-6)
	How satisfied were you with the technology of videoconferencing, for example, was the sound and picture clear?		6 (6-6)
	Would you recommend this form of treatment to someone else with diabetes?		6 (6-6)
	Would you recommend this form of treatment for other specialty care services?		6 (6-6)
**Primary care provider satisfaction, median (interquartile range)** ^**a**^			
	How would you rate your overall satisfaction with the diabetes telemedicine program?		5 (4-6)
	I value the information from the study endocrinologist		5 (5-6)
	This telemedicine service affords increased access to patient referrals to specialty areas		5 (5-6)
	My patients with diabetes appreciate that I can also offer specialty services		5 (3-6)
	The endocrinologist and I were able to effectively communicate a treatment plan together		5 (4-6)
	I received timely information from the endocrinologist		6 (4-6)
	Communication was passed between specialist and my office efficiently		6 (3-6)
	I was able to know my patients’ needs in the context of their diabetes		5 (4-6)
	I was assured that I would provide continuity of care to my patients with diabetes		6 (5-6)
	Our roles in the management of the patient were clear to me		5 (4-6)
	How likely would you be to use this program again?		5 (4-6)
	Would you recommend this form of program to other colleagues?		5 (4-6)
	Would you recommend this form of treatment for other specialty care services?		5 (4-6)

^a^ Likert score (range 0-6): 0 = very dissatisfied/do not recommend/highly disagree; 3 = neutral; and 6 = very satisfied/definitely recommend/highly agree.

Measurements of HbA1c after the teleconsultations (median 18 weeks) were available for 16 participants. In the other 9 participants, follow-up measurements were not available before the study was closed. At baseline, no participant (0/16) had an HbA1c < 7.0%, and 88% (14/16) had HbA1c ≥ 8.0% reflecting very poor glycemic control. After the teleconsultations, the proportion of patients with HbA1c ≥ 8.0% substantially decreased from 88% to 50% (*P* = .03, McNemar test). Mean HbA1c improved from 9.6 ± 0.4% to 8.5 ± 0.4% (*P* < .001, paired *t* test). Three-quarters of patients (75%, 12/16) experienced an absolute decrease in HbA1c of at least 0.5% from baseline.

## Discussion

### Main Findings

There is a disparity in diabetes care between rural and urban areas in the United States [1-3]. Although this disparity has several causes, access to physician care is an important contributor. Limited access to specialists is especially problematic [4]. Teleconsultations offer the potential to enhance the availability of diabetologist services to these communities. However, evidence that teleconsultations are well received by patients and providers in rural communities has not been demonstrated for diabetes management, which has its own complexities of care. Acceptability of this technology and care model is critical in the increasingly patient-centered environment where satisfaction and accountability take on greater importance.

Our findings demonstrate that videoconferencing-based teleconsultations specifically tailored to diabetes care are well received by both patients and primary care providers in a rural community. The direct implication of this finding is that broader adoption of this model is unlikely to be constrained by rejection of videoconferencing technology in the medical office, or primary care provider’s dissatisfaction with coordinated care delivered remotely. However, since this study targeted an underserved community, it is possible that responses were influenced by the fact that teleconsultations delivered previously unavailable care. Although such potential bias cannot be ruled out, it does not detract from the value of teleconsultations as an acceptable means of delivering care to rural communities.

How generalizable are the results to other rural communities? In our sample, age and gender was representative of typical patients with Type 2 diabetes in rural communities. Reluctance and/or inability to travel was highly prevalent, supporting generalization to other rural communities. However, race was homogenous and representative of only certain US rural populations. The number of rural primary care providers in the study was modest, but not surprising since the study targeted a rural community with physician shortages; therefore, this number is typical and reflective of such shortages. In terms of referral patterns, it is possible that primary care providers might have unconsciously referred those patients who were more open-minded about teleconsultations. Nevertheless, providers make decisions about referrals based on the likelihood patients will follow their referral recommendations; therefore, our study replicates real-world clinical referral patterns.

Although this study focused primarily on satisfaction, as an exploratory analysis we thought it would be worthwhile to examine whether teleconsultations had an influence on HbA1c levels. It is difficult to answer this question with absolute certainty because there was no control group. Despite this limitation, the improvements in HbA1c in the limited dataset were quite substantial and agree with the hypothesis that teleconsultations can deliver clinically meaningful results. One limitation in our findings is that follow-up HbA1c was not available for all participants before study closure. However, the missing data should not be construed as a sign of lack of satisfaction, since suboptimal adherence to frequent testing is also affected by barriers to care. Patients in rural communities often face a number of challenges in this regard. In fact, during teleconsultation visits, participants reported economic and transportation challenges to adhere to frequent testing and medical appointments.

### Comparison With Other Work

Despite the increasing attention on diabetes in our current health care environment, to our knowledge the only study examining specialist diabetes care delivered through video teleconsultation is the Informatics for Diabetes and Education Telemedicine (IDEATel) study [7]. The overall high satisfaction observed in our study is consistent with the IDEATel study, which examined a related, but different, model of telemedicine services offered by nurse case managers to patients with diabetes in urban and upstate New York. Differences between the type of telemedicine services in IDEATel and those in our study make direct comparisons inappropriate. Nonetheless, our findings complement the IDEATel study in supporting the notion that telemedicine technologies are well accepted in the context of diabetes care. Regrettably, at the time of our study, a validated questionnaire specific to diabetes telemedicine was unavailable and our sample size limited our ability to perform sound reliability testing on our survey. However, subsequent to our study, a diabetes telemedicine survey has been validated and our questions are consistent with that survey [7].

### Conclusions and Future Directions

In summary, we have demonstrated that a new model combining teleconsultations by an endocrinologist and local diabetes education supported by a nurse offer a potential solution to address the needs of diabetes care expertise often lacking in rural communities. Patients and primary care providers express a high degree of satisfaction, implying that adoption of the model is unlikely to be hampered by rejection of technology in the medical office or the approach by which services are provided. However, widespread adoption of this model will require demonstration of an unequivocal impact on glycemic control and barriers to funding will need to be addressed. There is a cost to implement the telecommunication infrastructure. However, with the shortage of endocrinologists in the United States [8], the costs may be lower than those needed to attract recruitment of endocrinologists to rural communities. Ultimately, the success of teleconsultations will depend on adequate compensation systems that would motivate physicians to provide teleconsultation services. Since this study began, some states and insurers have recognized the value of telemedicine and are beginning to reimburse for teleconsultations. For instance, the Pennsylvania Medical Assistance program revisited and expanded payment to include all physician specialists. They also revised the type of telecommunications technology that can be used to provide telemedicine consultation, specifically videoconferencing. Hopefully, compensation mechanisms like these will be further developed and spark broader implementation of teleconsultations as an alternative means of delivering diabetes specialty care to underserved communities.

## Abbreviations

AES: Advanced Encryption Standard
FIPS: Federal Information Processing Standard
HbA1c: glycosylated hemoglobin
IDEATel: Informatics for Diabetes and Education Telemedicine
